# Proinsulin and age in general population

**Published:** 2013-12-25

**Authors:** S Ateia, E Rusu, V Cristescu, G Enache, DM Cheța, G Radulian

**Affiliations:** * ”Carol Davila” University of Medicine and Pharmacy, Bucharest, Romania; ** Medas Clinic, Bucharest, Romania; *** ”Titu Maiorescu” University, Bucharest, Romania; **** Emergency Hospital Călărași, Romania; ***** ”Nicolae Paulescu” National Institute of Diabetes, Nutrition and Metabolic Diseases, Bucharest, Romania

**Keywords:** aging, insulin resistance, obesity, C-peptide, proinsulin

## Abstract

Abstract

Objective. The objective of this study was to assess the relationship between fasting proinsulin (PI) and age in general population and to determine whether there are differences regarding this association in obese and non-obese persons.

Methods. A random population-based sample (n=656) of Romanians (26–80 years) living in Bucharest, Romania was studied; 432 persons had diabetes and they were not analyzed in this paper.

Circulating levels of fasting plasma glucose (FPG), fasting plasma insulin (FPI), fasting plasma proinsulin (FPP), fasting plasma C-peptide, HbA1c, lipid profile, creatinine, urea were measured. The homeostasis model assessment of insulin resistance (HOMA-IR), HOMA-B, and Quicki index were also calculated.

Results. For all participants proinsulin was the highest in the third quartile of the age group (59-67 years), with a median proinsulin of 5.8 pmol/L. Subsequently, proinsulin increased with age, from 2.6 pmol/L for participants aged 20-51 years, to 4.7 pmol/L for participants aged 51-59 years; proinsulin levels decreased in the upper quartile 4.8 pmol/L for those aged over 67 years. In sex-specific analyses, proinsulin increased with age for both men and women, except for those in the upper quartile.

The prevalence of the obesity was 30.4% (n=68); obesity prevalence did not increase with age (p=0.26). Fasting proinsulin levels significantly increased with body mass index (BMI) category from lean (n=67, 2.9 pmol/L) to overweight (n=89, 4.5 pmol/L) and obese (n=69, 6.63 pmol/L) (p<0.0001).

Conclusions. Our study has demonstrated a close association between age and elevated proinsulin and proinsulin/insulin ratio in general population.

Abbreviations: BMI – body mass index, BP – blood pressure, DM – diabetes mellitus, eGFR– estimated glomerular filtration rate, FPI – fasting plasma insulin, FPG – fasting plasma glucose, FPP– fasting plasma proinsulin, HbA1c – glycosylated hemoglobin, HOMA-IR – the homeostasis model assessment of insulin resistance, HOMA-B – the homeostatic model for assessment of B-cell function, IGT – impaired glucose tolerance, MetS – metabolic syndrome, PIR – proinsulin to insulin ratio, WC – waist circumference, HDL-C – high density lipoprotein cholesterol, TG – triglycerides, TC – total cholesterol, T2DM – type 2 diabetes.

## Introduction

Aging is associated with important changes in metabolism and body composition [**1**]. Between 20 and 70 years of age, there is a progressive decrease of fat-free mass (mainly muscle) of about 40% and an increase of the fat mass. There is a relatively greater decrease in peripheral fat-free mass compared to central fat-free mass. After the age of 70 years, fat-free mass and fat mass decrease in parallel. Fat distribution changes with age so there is an increase in visceral fat, which is more pronounced in women than in men. Also, there is an increasing intramuscular (in the skeletal muscle) and intrahepatic fat deposition. Increased intramuscular and intrahepatic fat contributes to impaired insulin action through locally released adipokines and fat-free fatty acids. Increased pancreatic fat with declining β-cell function also plays a role. [**2**]

Proinsulin is a prohormone with low metabolic activity compared with mature insulin. In humans, insulin and proinsulin concentrations are significantly correlated with birth weight [**3**] and with various body measures including weight, head circumference [**4**].

The prevalence of abnormal glucose tolerance and diabetes mellitus increases with age [**5**]. Insulin release is reported to diminish with increasing age [**6**]. This decrease in insulin release could involve a reduction in islet mass, but could also be caused by a functional impairment of the beta cells with ageing. A decline in post-challenge insulin levels with advancing age has been observed in population studies [**7**]. Although this finding could reflect β-cell failure in ageing, it might also be due to alterations in diet or gastric emptying, or even to an enhancement in insulin sensitivity in older age. Although proinsulin levels increase together with insulin concentrations in insulin resistance [**8**], a raised ratio of proinsulin to insulin, due to a disproportionate release of proinsulin from β-cell, is considered an early marker of islet dysfunction [**9**]. In a cross-sectional study, TromsØ Study, a decline in random casual concentrations of insulin across increasing age groups in men, but not in women was observed [**10**].

The objective of this study was to assess the relationship between fasting plasma proinsulin (FPP) and age in general population and to determine whether the association is different in obese and non-obese persons.

## Materials and Methods

**Study population and sampling methods**

**Design**

Cross-sectional population-based screening campaign whose main objective was the screening for diabetes.

**Subjects**

Patient recruitment took place in November-December 2011 in Bucharest, Romania. During this campaign a total of over 15,000 people were assessed. Only data from patients who gave their consent were analyzed and processed. A random population-based sample (n=656) of Romanians (26–80 years) was studied; 432 persons had diabetes and they were not analyzed in this paper.

The exclusion criteria were: patients with a previous diagnosis of diabetes, pregnancy, patients having an alcohol consumption of more than 20 g/day for women and 30 g/day for men, history of pancreatitis, chronic liver disease, autoimmune liver disease, hemochromatosis, HIV infection, patients with history of hepatotoxic or steatosis-inducing drug use, currently on interferon treatment or during the last 12 months, recent surgery, inflammatory or malignant disease, anticoagulant therapy, steroid therapy, postmenopausal women on estrogen replacement therapy.

**Measurements**

**Clinical examination**

Clinical examination was performed according to the principles of medical ethics standards and included the following parameters: height, weight, waist circumference, hip circumference, systolic and diastolic blood pressure measured in one arm, after ten minutes rest.

Body mass index (BMI) was calculated (body weight in kilograms divided by the square of height in meters) and categorized based on national guidelines. Participants whose average blood pressure levels were greater or equal to 140/90 mmHg or using antihypertensive medication were classified as hypertensive subjects. [**11**] Metabolic syndrome (MetS) was diagnosed according to IDF criteria [**12**].

**Laboratory assays**

Fasting blood samples were drawn between 7:00 a.m. and 10:00 a.m. The biochemical analyses, including fasting plasma glucose (FPG), fasting plasma insulin (FPI), fasting plasma proinsulin (FPP), fasting plasma C-peptide, HbA1c, total cholesterol (TC), triglycerides (TG), high-density lipoprotein cholesterol (HDL-C), creatinine, urea, were measured after an overnight fasting period of 12h, using routine clinical chemistry methods and then documented.

Intact proinsulin was measured by using ELISA (Demeditec Diagnostics GmbH, Germany). The inter- and intraassay CVs was 4.3% and 5.5% for proinsulin. Serum insulin and C-peptide were determined by chemiluminescence enzyme immunoassay (Architect, Abbott). The cross-reactivity of insulin with proinsulin, and with C-peptide was 0.1% respectivelly 0.001%.

IR (insulin resistance) was determined by using the Homeostasis model assessment (HOMA-IR) (fasting insulin level (mUI/l)x fasting glucose level (mg/dl)/405 [**13**]; a HOMA-IR index value of more than 2.0 was considered as the criteria of insulin resistance. The homeostatic model for assessment of B-cell function (HOMA-B) was calculated by using the formula [**13**]: 20×FPI (μU/ml)/(FG (mmol/l) – 3.5). The quantitative insulin sensitivity check index (QUICKI) was calculated by using the formula: 1/(log (FPI) (μU/ml)+log (FPG) (mg/dl)) [**14**].

Chronic kidney disease (CKD) was characterized as an estimated glomerular filtration rate (eGFR) <60 mL/min/1.73m2 by using CKD-EPI formula, which has been shown to be more accurate than the MDRD formula for classifying individuals as having CKD [**15**].

**Statistical analysis**

The statistical analysis was performed by using SPSS 19 (copyright IBM). The cohort was divided into the following four groups based upon the age quartiles: 20–51, 52–59, 60-67, and 68–85 years. Results were reported as means and standard deviation for continuous variables normally distributed, and % for dichotomy data. The one-way analysis of variance and Chi square and Fisher exact tests analyses were used to compare the means and proportions, respectively were used to test the differences in proportions. The correlations between the different parameters were estimated by using the Spearman correlation coefficients. No multiplicity adjustments were used as no intermediate analysis was performed. Type I error assumed was of 5%.

## Results

We have included 224 patients, 137 women (61.2%) and 87 men (38.8%) with a mean age of 57.3±14.1 years, range 20–85 years. Characteristics of the subjects included in the study are summarized in (Table 1). The average (mean±SD) BMI was 27.7±4.9 kg/m², the mean±SD systolic blood pressure was 142±20.6 mmHg. The mean±SD for total and HDL cholesterol was 217.8±47.5 mg/dL and 55.3±13.7 mg/dL, respectively. Median (95% CI) concentrations for FPG, FPI, FPP, PIR were 100.5 (102.35-102.4 mg/dL), 8.59 μU/ml (9.33 – 9.37 μU/mL), 4.3 pmol/l (5.68-5.72 pmol/L), respectively 3.65 (4.91 – 4.96) and 1345.3 pg/mL (1317.9–1373.2 pg/mL). The median (95% CI) concentrations for C-peptide, HOMA-IR, HOMA-B were 2.13 ng/mL (2.4 - 2.41 ng/mL), 2.14 (2.39 - 2.4), and 84% (86.3-100.1%) respectively.

**Table 1:**
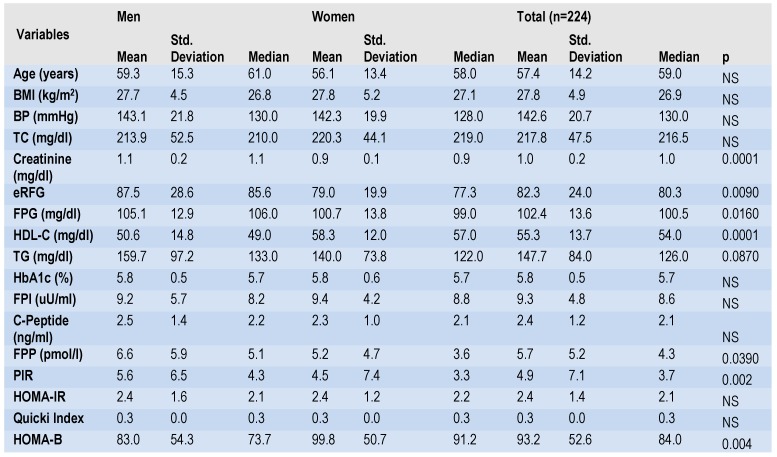
Clinical characteristics of patients

The prevalence of obesity in the entire lot was 30.4% (n=68), the prevalence of hypertension was 47.3% (n=106), the prevalence of hypercholesterolemia was 73.7% (n=165), the prevalence of hypertriglyceridemia was 34.8% (n=78), for metabolic syndrome the prevalence was 26.8% (n=60); no statistically significant differences were found regarding these these comorbidities and gender distribution. The analysis of each of these variables according to age showed a significant age trend for BMI, an increased prevalence of hypertension, hypercholesterolemia, metabolic syndome, HOMA-IR >2. HDL-C, TG, LDL-C did not show a significant trend with age.

**Proinsulin trends**

For all the participants, proinsulin was the highest in the third quartiles of the age group (59-67 years), with a median proinsulin of 5.8 pmol/L for that group. Subsequently, proinsulin increased with increasing age, from 2.6 pmol/L for those aged 20-51 years, to 4.7 pmol/L for those aged 51-59 years; proinsulin levels decreased in the upper quartile 4.8 pmol/L for those aged over 67 years (Table 2).

**Table 2:**
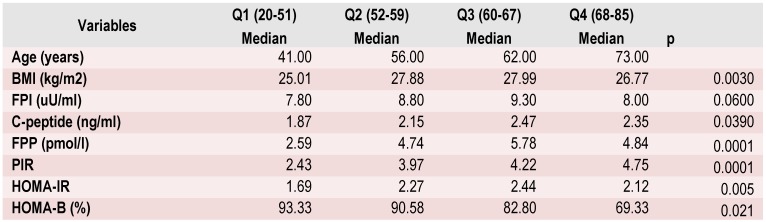
Clinical and laboratory characteristics of patients, stratified according to the age group

In sex-specific analyses, proinsulin increased with increasing age for both men and women, except for those in the upper quartile. The highest proinsulin of 6.46 was observed in men, in the age group 59-67 years, subsequently dropping to 4.58 pmol/l and 3.67 pmpl/l for 51-59 and 20-51 age group respectively. Similar results were observed in women; proinsulin in women started from 2.49 pmol/l in the age group of 20–51 years, then gradually increased thereafter to 4.79 pmol/l, 6.06 pmol/l, and it dropped to 3.77 pmol/l for the 68–85 age subcategory.

**Proinsulin and obesity**

The prevalence of the obesity was 30.4% (n=68), 25.3% in men (n=22) and 33.6% in women (n=46) (p=0.233); obesity prevalence did not increase with age (p=0.26). Fasting proinsulin levels significantly increased with body mass index (BMI) category from lean (n=67, 2.9 pmol/L) to overweight (n=89, 4.5 pmol/L) and obese (n=69, 6.63 pmol/L) (p<0.0001).

The ratio of proinsulin to insulin fasting levels did not differ significantly with BMI (p=0.95). The group with the highest FP quartiles showed a higher prevalence of obesity (n=26, 38.2%) (p < 0.003).

In univariate analysis, fasting proinsulin level was correlated both in women and men with weight, BMI, and waist circumference (Table 3).

**Fasting plasma proinsulin and metabolic syndrome**

The prevalence of the MetS was 26.8% (n=60), 27.6% in men (n=24) and 26.3% in women (n=36) (p=0.874) and it increased with age (p=0.01). The prevalence of the MetS was 45.6% (n=31) among obese (BMI ≥ 30 kg/m2) and 18.6% among non-obese participants (n=29). Fasting proinsulin levels significantly increased with components of MetS (p<0.0001), and this significance persisted (p = 0.0001) following adjustment for age and BMI or WC.

**The total proinsulin/insulin ratio**

PIR was significantly different between genders (4.3 pmol/L in men vs. 3.3 pmol/L in women, p=0.002). PIR increased with age (2.4 pmol/L for participants from the first quartile, 3.97 pmol/L in the second quartile, 4.2 pmol/L in the third quartile, and 4.7 pmol/L in the fourth quartile) (p=0.005). There were no differences according to the degree of obesity, presence of obesity, or metabolic syndrome. The total proinsulin/insulin ratio increased with age, hypertension, triglycerides, higher fasting glucose concentrations, Hba1c, but there was no significant relationship between PIR and waist circumference, BMI, HOMA-IR (Table 3).

**Table 3:**
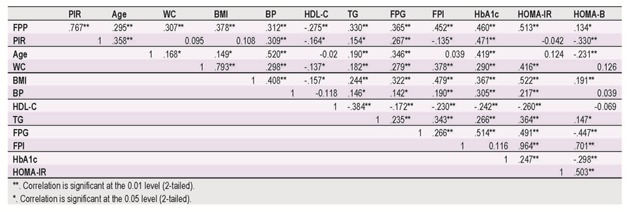
Spearman correlation coefficient between proinsulin and anthropometric and metabolic parameters

**Fasting plasma proinsulin and kidney function**

There were no statistically significant differences between the FPP, PIR, FPI, C-peptide and estimated glomerular filtration rate (all p>0.05). If eGFR was stratified as under or above 60 mL/min/1.73 m² we found significantly higher FPI levels in persons with eGRF above 60 mL/min/1.73 m² (2.16 uU/mL vs. 1.9 uU/mL p=0.05); there were no differences for FPP, PIR, C-peptide.

## Discussions

This paper highlighted that proinsulin levels and proinsulin-to insulin ratio increased across age groups in both men and women. Fasting insulin levels have been reported to increase [**16**] or not to change [**17**] with age in cross-sectional studies. There were no statistically significant changes in FPI in our study. In elderly persons, the Baltimore Longitudinal Study of Aging [**18**] and the Hoorn Study [**19**] reported a lower post-challenge insulin response. Hyperproinsulinaemia relative to insulin [**20**] indicates impaired proinsulin processing, which is an early abnormality of beta cell function. Proinsulin concentrations relative to insulin are increased in both impaired glucose tolerance (IGT) [**21**] and type 2 diabetes (T2DM) [**22**]. Longitudinal data in older persons with IGT also indicate that the proinsulin-to insulin ratio is a marker for progression to diabetes [**23**].

Fasting proinsulin levels significantly increased with BMI category from lean (n=67, 2.9 pmol/L) to overweight (n=89, 4.5 pmol/L) and obese (n=69, 6.63 pmol/L) in our study but the ratio of proinsulin to insulin fasting levels did not differ significantly with BMI. The group with the highest FP quartiles showed a higher prevalence of obesity. Obesity in most subjects with T2DM [**24**] seems to be also involved in the occurrence of hyperproinsulinemia. Univariate results from previous papers have indicated that proinsulin concentrations were elevated in obese subjects [**25**-**27**] and proinsulin was positively correlated with BMI and WHR [**28**,**29**]. Similarly, in our study, in univariate analysis, fasting proinsulin level correlated both in women and in men with weight, BMI, and waist circumference.

Hyperinsulinemia and hyperproinsulinemia in obese patiets can be explained as a reflecting compensatory hyperfunction of β-cells in the presence of presumable insulin resistance due to obesity. Normal glucose tolerance in these obese subjects would be interpreted as the result of physiological hyperfunction of pancreatic β-cells to overcome insulin resistance.

It seemed that absolute and relative hyperproinsulinemia in elderly subjects was caused by β-cell dysfunction in processing proinsulin to insulin, since there was no evidence of β-cell hypersecretion and/or altered clearance of insulin and C-peptide. It has also been reported that insulin is metabolized mainly in the liver and C-peptide and proinsulin are degraded in the kidney. The possibility of clearance changes of peptides may exist in old age. It seems unlikely, however, that the results are attributable only to altered clearance of proinsulin and not of insulin or C-peptide in elderly people [**30**,**31**].

Because the fraction of total metabolic clearance accomplished by the kidneys is greater for proinsulin than for insulin [**32**], an age-related decline in renal function could conceivably have led to increased proinsulin concentrations relative to insulin, as we observed with advancing age. Advanced renal damage may also in itself affect glucose metabolism and both may cause insulin resistance and impaired insulin secretion [**33**].

## Conclusions

In conclusion, in sex-specific analyses, proinsulin increased with advancing age for both men and women, except those in the upper age quartile. This paper reinforces the observation of elevated proinsulin concentrations among subjects with obesity.
